# A case report of synchronous bilateral breast cancer with distinct histological subtypes and favorable long-term survival

**DOI:** 10.3389/fonc.2026.1861415

**Published:** 2026-06-12

**Authors:** PingBo Zhang, DaWei Zhao

**Affiliations:** Department of Breast Surgery, Jilin Cancer Hospital, Changchun, Jilin, China

**Keywords:** apocrine metaplasia, individualized treatment, luminal breast cancer, mucinous carcinoma, multidisciplinary tumor board, multiple primary malignant tumors, synchronous bilateral breast cancer, triple-negative breast cancer

## Abstract

Bilateral synchronous breast cancer accounts for a small proportion of all breast cancer cases, and those with markedly different pathological and molecular subtypes are extremely rare. Such cases pose substantial challenges in accurate diagnosis, treatment decision-making, and prognostic evaluation. Here, we present a 51-year-old postmenopausal female patient who presented with an incidental painless left breast mass. Imaging examinations revealed multiple bilateral breast lesions classified as BI-RADS 4A, 4B, and 4C. All clinical and imaging data were reviewed by the multidisciplinary tumor board to develop personalized treatment regimens. Intraoperative frozen pathology verified bilateral breast malignancy, and the patient received bilateral mastectomy. Postoperative pathological examination confirmed two independent primary breast cancers: the right breast lesion was invasive breast carcinoma with apocrine differentiation showing a triple-negative immunophenotype, while the left breast contained multifocal mucinous carcinoma with a luminal-like immunophenotype. Subsequent adjuvant therapy included six cycles of TAC chemotherapy (liposomal doxorubicin, liposomal paclitaxel, and cyclophosphamide), followed by eight cycles of capecitabine maintenance therapy and long-term adjuvant endocrine therapy with exemestane. At 31 months postoperatively, the patient remained disease-free with no signs of local recurrence or distant metastasis. This case highlights the importance of comprehensive imaging evaluation, thorough pathological examination, accurate molecular subtyping, and individualized multidisciplinary treatment for patients with synchronous bilateral breast cancer of heterogeneous subtypes.

## 1 Introduction

Breast cancer exhibits distinctive epidemiological patterns and substantial biological heterogeneity, posing a persistent challenge to clinical management. The progressive delineation of molecular subtypes has refined our understanding of this disease and enabled increasingly precise therapeutic strategies. Nevertheless, marked intertumoral and intratumoral heterogeneity persists even within individual patients, complicating optimal clinical decision-making (1). Bilateral breast cancer, whether synchronous or metachronous, represents a compelling manifestation of such heterogeneity, with complexity further compounded when the two lesions present entirely divergent pathological subtypes.

In addition to bilateral breast involvement, there are also multiple primary tumors co-occurring in the breast and other organ systems (2–5). These malignancies share common risk factors including advanced age, genetic susceptibility, chronic inflammatory stimulation, and unhealthy lifestyle, leading to overlapping high-risk populations and multifaceted pathogenic mechanisms (2, 6). In a single-center retrospective analysis of 15,321 patients with malignancies in China, Zhai et al. reported that the prevalence of multiple primary malignant tumors was 1.09%, with digestive system malignancies being the most frequently involved site, followed by lung cancer. Breast cancers accounted for 6.59% of first primary malignancies and 4.19% of second primary malignancies, representing a notable proportion among the affected organs (7). Iorga et al. identified 15 cases of synchronous breast and colorectal malignancies, with colorectal cancer diagnosed first in nine cases and breast cancer first in six. Invasive ductal carcinoma was the most common breast subtype, while all colorectal tumors were adenocarcinomas. Surgical resection was indicated for both malignancies in 14 cases, with four undergoing concomitant colectomy and mastectomy (8). These findings highlight that synchronous breast and other malignancies require comprehensive diagnostic evaluation and individualized therapeutic strategies, with multidisciplinary tumor board consultation being essential for clinical decision-making.

Synchronous bilateral breast cancer is generally defined as the diagnosis of malignant tumors in both breasts simultaneously or within 6 months of each other (9). Although it accounts for only 1%–5% of all breast cancers, its management remains challenging due to frequent discordance in tumor origin, histology, molecular phenotype, and biological behavior between the two breasts (9–11).

Apocrine carcinoma of the breast is a rare histological subtype, accounting for less than 1% of all breast cancers (12). It is characterized by abundant eosinophilic cytoplasm and distinct apocrine differentiation and frequently demonstrates an estrogen receptor (ER)-negative, progesterone receptor (PR)-negative, and androgen receptor (AR)-positive immunophenotypes overlapping with the triple-negative breast cancer (TNBC) (13). Mucinous carcinoma, also known as colloid carcinoma, is another rare subtype characterized by abundant extracellular mucin production, relatively indolent biological behavior, and a favorable prognosis (14). It is characteristically ER-positive with a luminal-like molecular features. The synchronous presentation of these two rare and biologically distinct subtypes in bilateral breasts has rarely been documented (15).

In this report, we describe a 51-year-old postmenopausal woman with synchronous bilateral breast cancer, specifically invasive apocrine carcinoma in the right breast and multifocal mucinous carcinoma in the left. The two tumors exhibited markedly different immunohistochemical profiles with respect to hormone receptor status, HER2 status, and Ki-67 proliferation index. The patient received a tailored multidisciplinary treatment comprising surgery, adjuvant chemotherapy, maintenance chemotherapy, and long-term endocrine therapy, achieving favorable disease control on follow-up. We discuss the clinical, radiological, pathological, and therapeutic characteristics of this rare case to inform clinical practice regarding the diagnosis and management of heterogeneous synchronous bilateral breast cancer.

## 2 Case presentation

A 51-year-old postmenopausal woman was admitted after incidentally detecting a small, painless mass in the upper outer quadrant of the left breast two days prior. The mass was small, non-tender, and without skin changes, nipple retraction, or discharge. The patient reported no systemic symptoms, including fever, weight loss, fatigue, or night sweats. Her past medical history was otherwise unremarkable, with no chronic diseases, prior surgeries, blood transfusions, drug or food allergies, and no smoking or alcohol consumption. Her menstrual history showed menarche at age 14 and natural menopause at age 49, with gravida 2, para 2 and no hormonal replacement therapy. No family history of malignancy was found among first and second-degree relatives, and no hereditary cancer syndrome was suspected.

After admission, physical examination revealed no abnormalities. Comprehensive preoperative imaging was subsequently performed, including bilateral breast ultrasound and magnetic resonance imaging (MRI). Ultrasound demonstrated multiple bilateral lesions: a mass in the left upper outer quadrant was categorized as BI-RADS 4B, a mass in the right inner upper quadrant as BI-RADS 4A, and additional nodules in both breasts as BI-RADS 3 (**Figure 1**). Breast MRI characterized these findings, further revealing irregular, malignant-suspicious masses in the left upper outer quadrant and right inner lower quadrant (BI-RADS 4C), with additional small suspicious foci and scattered benign-appearing nodules bilaterally (**Figure 2**). These findings were consistent with bilateral multifocal malignancy and indicated the necessity of surgical intervention.

**Figure 1:**
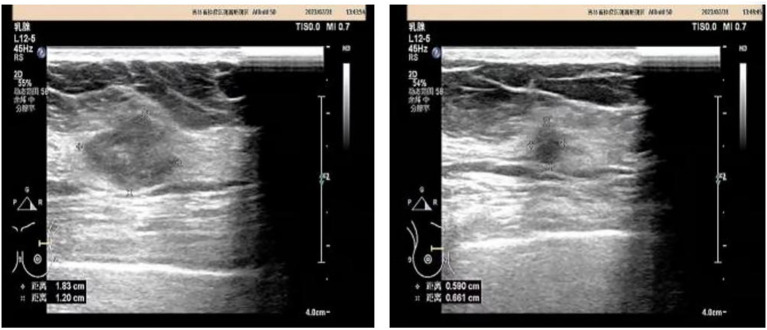
Breast ultrasound showing multiple bilateral breast lesions. A mass in the upper outer quadrant of the left breast was classified as BI-RADS 4B, a mass in the inner upper quadrant of the right breast as BI-RADS 4A.

**Figure 2:**
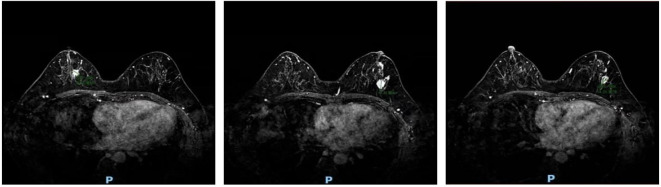
Breast magnetic resonance imaging (MRI) revealing irregular masses in the inner lower quadrant of the right breast and upper outer quadrant of the left breast, both categorized as BI-RADS 4C. A smaller lesion in the left lower outer quadrant was rated BI-RADS 4A.

Given the reliability of imaging findings and the small size of multifocal bilateral lesions, intraoperative frozen section was empirically employed to confirm bilateral malignancy and guide immediate surgical decision-making. After completing preoperative examinations and excluding surgical contraindications, the case was presented to our multidisciplinary tumor board (MDT), comprising breast surgery, medical oncology, radiology, and pathology, for comprehensive evaluation. Considering the absence of preoperative pathological results, the MDT team proposed a tentative clinical diagnosis of suspected synchronous bilateral breast cancer and formulated a personalized management strategy centered on surgical resection, with postoperative adjuvant treatment to be determined based on final intraoperative and postoperative pathological outcomes.

On August 3, 2023, the patient initially underwent bilateral breast segmental resection for intraoperative diagnosis. Following confirmation of malignancy, bilateral total mastectomy and bilateral sentinel lymph node biopsy were performed under general anesthesia, as per the MDT consensus. The procedure was smooth without intraoperative complications. All surgical specimens were sent for intraoperative rapid pathological examination and postoperative routine pathological examination.

Postoperative pathological examination confirmed two independent primary breast cancers with divergent histological subtypes. The right breast lesion was invasive apocrine carcinoma (grade II), accompanied by a 10% high-grade ductal carcinoma *in situ* component, measuring 0.9 cm in maximum diameter. Three right axillary sentinel lymph nodes were negative for metastasis (0/3). Immunohistochemistry demonstrated ER (–), PR (–), AR(40%+), HER2(2+, FISH-negative), and Ki-67(10%), consistent with triple-negative apocrine carcinoma (**Figure 3**).

**Figure 3:**
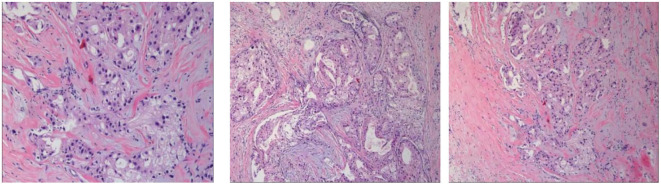
Paraffin-embedded pathology of the right breast tissue showing invasive breast carcinoma with apocrine differentiation.

The left breast harbored two separate mucinous carcinoma foci measuring 1.6×1.5×1.2 cm and 1.2×1.2×0.9 cm, respectively, both with infiltrative borders. Peritumoral lymphovascular invasion was present; perineural invasion was absent. Stromal tumor-infiltrating lymphocytes (TILs) were less than 5%. Five left axillary sentinel lymph nodes were harvested, with one showing isolated tumor cells (1/5). Immunohistochemistry of the two foci demonstrated heterogeneous luminal-like phenotypes with high ER expression, discordant Ki-67 proliferation indexes, and negative HER2 amplification, supporting their clonal independence. All surgical margins (nipple, skin, superficial, deep, and basal) were negative for carcinoma, indicating complete resection (**Figure 4**).

**Figure 4:**
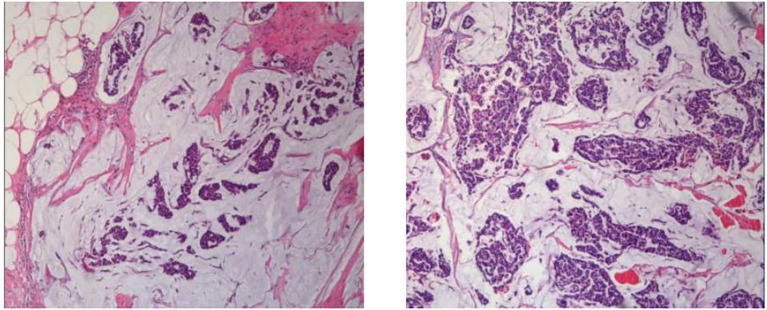
Paraffin-embedded pathology of the left breast tissue showing two separate lesions, both consistent with mucinous carcinoma.

Based on the postoperative pathological results, molecular subtypes, adverse pathological features, and the patient’s physical status, the MDT team optimized the adjuvant treatment scheme. The patient received six cycles of TAC adjuvant chemotherapy, with mild and manageable adverse effects after symptomatic treatment. Following chemotherapy, eight cycles of oral capecitabine maintenance therapy were administered for consolidation. Given the patient’s postmenopausal status and ER-positive status of the left mucinous carcinoma, long-term adjuvant endocrine therapy with exemestane was continued.

The patient underwent regular follow-up every three months postoperatively, including physical examination, tumor marker assessment, and thoracoabdominal imaging. Annual bone scan surveillance was also performed. At 31 months after surgery, the patient remains free of local recurrence, regional lymph node metastasis or distant organ metastasis, with stable general condition and good quality of life.

## 3 Discussion

Synchronous multiple primary malignant tumors have become an increasingly recognized clinical entity in modern oncology. The widespread adoption of high-resolution imaging modalities and prolonged life expectancy have contributed to their rising detection (16). Unlike metastatic lesions, synchronous multiple primary tumors arise from independent clonal origins, exhibiting distinct biological behaviors, diagnostic criteria, and therapeutic principles, rendering accurate differentiation central to clinical management (17–19). Chen et al. reported 31 patients with synchronous primary breast and lung cancer; 24 presented with breast cancer first and were diagnosed with lung cancer by routine chest computed tomography (CT), while 7 presented with lung cancer first and were found to have synchronous breast cancer by positron emission tomography (PET)-CT. All patients underwent surgery, and survival analysis demonstrated comparable outcomes between these synchronous cases and matched patients with lung cancer alone. These findings highlight the importance of comprehensive imaging evaluation and multidisciplinary coordination in managing synchronous primary tumors. Particularly, for patients requiring preoperative systemic therapy, the regimen should be selected to balance the treatment of each malignancy (20). Zheng et al. analyzed 87,752 female breast cancer patients and reported that 17.0% developed second primary cancers (SPCs), with second breast cancer accounting for 57.9% of these cases. Familial predisposition significantly increased the risk of 14 site-specific SPCs; among these, second ovarian cancer showed the highest risk (RR = 6.28). Importantly, the fatality rate in patients with SPC was twice that of those without SPC (32.3% vs. 16.1%), and SPCs were the leading cause of death (64.9%). These data underscore that breast cancer survivors harbor a substantial long-term risk of additional primary malignancies, highlighting the importance of extended surveillance and familial risk assessment in long-term follow-up.

Synchronous bilateral breast cancer with entirely divergent histological and molecular subtypes is an exceptionally rare clinical entity (15). The present case exemplifies this diagnostic challenge, with invasive apocrine carcinoma in the right breast and multifocal mucinous carcinoma in the left. Distinguishing such synchronous dual-primary lesions from metastatic carcinoma is fundamental, as metastatic disease typically demonstrates concordant histological and immunophenotypic features, whereas independent primaries exhibit divergent profiles (21). Comprehensive imaging, thorough immunohistochemical evaluation, and multidisciplinary team consultation are therefore essential to establish this differential diagnosis and guide individualized treatment (22).

Apocrine carcinoma of the breast is a rare subtype with unique morphological and immunophenotypic features. It is frequently ER-negative and PR-negative but often expresses AR, which represents a potential therapeutic target (13). Despite its triple-negative profile, apocrine carcinoma may demonstrate a more favorable prognosis than conventional high-grade triple-negative breast cancer, especially when the Ki-67 proliferation index is low (23). In the present case, the right-sided apocrine carcinoma showed a Ki-67 index of 10%, suggesting relatively low proliferative activity, which may have contributed to the favorable outcome.

Mucinous carcinoma of the breast is generally considered an indolent subtype with a favorable prognosis due to its slow growth and low metastatic potential (14). However, adverse pathological features, such as lymphovascular invasion and lymph node metastasis, may increase the risk of recurrence. In this case, the left-sided mucinous carcinoma showed lymphovascular invasion and tumor cell metastasis in one sentinel lymph node, indicating the necessity of adequate adjuvant systemic therapy. The high ER expression supported the use of adjuvant endocrine therapy to reduce long-term recurrence risk (24).

The coexistence of these two biologically divergent subtypes necessitated a lesion-specific multidisciplinary strategy. Adjuvant TAC chemotherapy was administered for the triple-negative apocrine carcinoma (25), followed by capecitabine maintenance therapy. Concurrently, endocrine therapy with exemestane was administered to target the ER-positive carcinoma (26). This individualized approach achieved 31 months of disease-free survival.

Some key clinical lessons can be drawn from this case. First, for patients with bilateral breast lesions detected on imaging, comprehensive evaluation including ultrasound and MRI is necessary to identify synchronous malignant lesions. Second, thorough pathological examination with complete immunohistochemical and FISH testing is essential to distinguish dual-primary tumors from metastatic disease and to determine molecular subtypes. Third, treatment should be individualized according to the most aggressive subtype and all adverse pathological features (27). Fourth, long-term regular follow-up is crucial for early detection of recurrence and timely intervention.

Several limitations should be acknowledged. This is a single case with a limited follow-up period of 31 months. Longer follow-up is required to evaluate late recurrence and long-term survival. In addition, next-generation sequencing (NGS) was not performed to further explore the genomic characteristics of the two tumors. Future studies with larger sample sizes and genomic profiling may improve our understanding of heterogeneous synchronous bilateral breast cancer.

## 4 Patient perspective

The patient maintained a positive attitude throughout diagnosis and treatment. She reported good tolerance to surgical and adjuvant chemotherapy, with manageable and transient adverse reactions. Regular follow-up examinations alleviated anxiety and provided reassurance regarding disease status. She adhered to oral endocrine therapy as prescribed, maintaining a stable daily routine and favorable quality of life. The patient expressed satisfaction with the multidisciplinary treatment and long-term disease control.

## 5 Conclusion

We report a rare case of synchronous bilateral breast cancer with two distinct histological subtypes: invasive apocrine carcinoma with a triple-negative phenotype in the right breast and multifocal mucinous carcinoma with luminal phenotype in the left breast. Comprehensive imaging, accurate pathological diagnosis, and molecular subtyping enabled appropriate surgical intervention and individualized multidisciplinary treatment, including chemotherapy, maintenance therapy, and long-term endocrine therapy. Individualized MDT decision-making provided crucial support for accurate diagnosis and precise treatment of this rare case. The patient achieved favorable disease control and remained disease-free at 31 months postoperatively. This case highlights the clinical importance of recognizing heterogeneous synchronous bilateral breast cancer and implementing tailored treatment strategies to optimize outcomes.

## Data Availability

The original contributions presented in the study are included in the article/supplementary material. Further inquiries can be directed to the corresponding author.
